# Supplementary material for: High Carbon Dioxide Concentration Inhibits Pileus Growth of Flammulina velutipes by Downregulating Cyclin Gene Expression
Source: J Fungi (Basel). 2025 Jul 24;11(8):551. doi: 10.3390/jof11080551 (PMC12387923; doi:10.3390/jof11080551)
Supplement: Supplementary file 1 [file jof-11-00551-s001.zip › Figure S1.pdf]

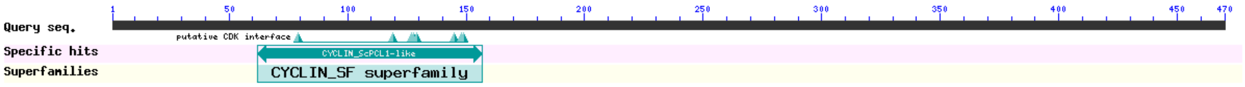

Pssm-ID: 410260 **Cd Length:** 94 **Bit Score:** 89.25 **E-value:** 1.17e-21

```
                         10        20        30        40        50        60        70        80        90
                 ....*....|....*....|....*....|....*....|....*....|....*....|....*....|....*....|....*....|....*.
DN1560_c0_g1_i9  62 SLSKFIVRLLSQCNISTATLLTCLIYFDRLRAKMSHVHHATESAMHRVFFATLIVARKYLNDSSPKNVHWAAYAVgyFDLAEINTMEHQLLKVLNF 157
Cdd:cd20557       1 SLRSFIARLLRRTRLSPSVVLLALVYLERLRRRLPEALLGQEGSPYRLFLVALMLASKYLDDESYSNKSWAEISG--LPVRELNAMEREFLEALDW 94
```

DN1560_c0_g1_i9

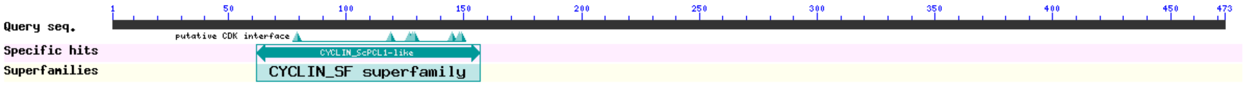

Pssm-ID: 410260 **Cd Length:** 94 **Bit Score:** 93.49 **E-value:** 3.31e-23

```
                         10        20        30        40        50        60        70        80        90
                 ....*....|....*....|....*....|....*....|....*....|....*....|....*....|....*....|....*....|....*.
DN6954_c0_g1_i2  62 SLSRFIVRIVKQVHISTATLLSCLIYFDRLRIKMSRLHQASETAMHRVFFATLIVASKYLNDSSPKNVHWAAYAveTYDLVEINLMERQLLHLLDF 157
Cdd:cd20557       1 SLRSFIARLLRRTRLSPSVVLLALVYLERLRRRLPEALLGQEGSPYRLFLVALMLASKYLDDESYSNKSWAEIS--GLPVRELNAMEREFLEALDW 94
```

DN6954_c0_g1_i2

Figure S1. Conserved domain analysis of two cyclin genes identified under elevated CO_2 conditions in *F. velutipes*. NCBI Conserved Domain Database (CDD) search results showing the presence of conserved cyclin_N box domains at the N-termini of DN1560_c0_g1_i9 and DN6954_c0_g1_i2. The alignment highlights regions of high sequence similarity to the cyclin_SF superfamily, particularly PHO85-type cyclins from *Saccharomyces cerevisiae*.
