# Supplementary material for: High Carbon Dioxide Concentration Inhibits Pileus Growth of Flammulina velutipes by Downregulating Cyclin Gene Expression
Source: J Fungi (Basel). 2025 Jul 24;11(8):551. doi: 10.3390/jof11080551 (PMC12387923; doi:10.3390/jof11080551)
Supplement: Supplementary file 1 [file jof-11-00551-s001.zip › Figure S2.pdf]

	10 20 30
DN6954_c0_g1_i2_F.velutipes	DLVEINLMERQLLHLLDFDLRFTEEEACFHFAP
DN1560_c0_g1_i9_F.velutipes	DLAEINTMEHQLLKVLNFDLRFTEEDACLHFAP
Cylindrobasidium torrendii FP1	DLAEINTMEHQLLKVLNFDLRFTEEDACLHFAP
Armillaria borealis	PVAEINLMERQLLHLLDFDLRFSEDDACRYFAP
Armillaria nabsnona	NVTEINLMEIQLLYLLDYDLRFSEEEACALFAP
Armillaria ostoyae	NVTEINLMEIQLLYLLDYDLRFSEEEACALFAP
Desarmillaria tabescens	NVTEINLMEIQLLYLLDYDLRFSEEEACALFAP
Desarmillaria tabescens	TVTEINLMEIQLLYLLDYDLRFNEEEACALFAP
Armillaria gallica	TVTEINLMEIQLLYLLDYDLRFNEEEACALFAP
Armillaria luteobubalina	NVTEINLMEIQLLYLLDYDLRLSEEEACALFAP
Armillaria fumosa	KVTEINLMEIQLLYLLDYDLRFSEEEACALFAP
Moniliophthora roreri MCA 2997	KVTEINLMEIQLLYLLDYDLRFSEEEACALFAP
Lentinula detonsa	PVSEINLMERQLLTLLDYDLRFDEEEACRYFAP
Lentinula raphanica	TIEEITLMETQLLGFLDYDLRFDEEEACRMFAP
Collybiopsis confluens	TIEEITLMETQLLGFLDFDLRFDEEEACRVFAP
Moniliophthora roreri	TKEEITLMETQLLGFLDFDLRFDEEEACRAFAP
Paramarasmius palmivorus	PVSEINLMERQLLTLLDYDLRFDEEEACRYFAP
Lentinula edodes	SMEEINLMERQLLALLNYDLRFDEEEACRYFAP
Marasmiellus scandens	TIEEITLMETQLLGFLDYDLRFDEEEACHMFAP
Lentinula aff. detonsa	PLKEINLMESELLNVLDFDLRFDEDEACRAFAP
Lentinula edodes	TIEEITLMETQLLGFLDYDLRFDEEEACRMFAP
Tetrapyrgos nigripes	TIEEITLMETQLLGFLDYDLRFDEEEACHMFAP
Mycena polygramma	PLNEINLMEAQLLSMLAFDLRFDEEEACKAFGH
Lentinula lateritia	QVSEVNLMEQQLLFLLDYDLRFDEEEACAAFAP
Marasmius fiardii PR-910	TIEEITLMETQLLGFLDYDLRFDEEEACRMFAP
Mycena latifolia	TLENVNIMERQLLELLDFNLRFNEEEACRYFAP
Desarmillaria ectypa	KVSEVNLMEQQLLFLLDYDLRFDEEEACAVFAP
Pluteus cervinus	TVTEINLMEIQLLYLLDYDLRFSEEEACALFAP
Mycena vitilis	DLEEVNLMERQLLGLLEYDLRFDELEACTYFAP
Mycena sp. CBHHK59/15	PVSEVNLMEQQLLFLLDYDLRFDEEEACAAFAP
Mycena belliarum	QVNEVNVMEQQLLFLLDYDLRFDEEEACAVFAP
Mycena maculata	KVGEVNLMEQQLLFLLDYDLRFDEEEACSAFAP
Mycena kentingensis (nom. inva	QVTEVNLMEQQLLFLLDYDLRFDEEDACVAFAP
Amanita brunnescens Koide BX00	QTSEVNLMESQLLFLLDYNLRFDEEIACRMFAP
Mycena galericulata	PIEEINLMEKQLLYLLDYDLRFDEADVCALFAP
Gymnopus androsaceus JB14	QVSEVNLMEQQLLFLLDYDLRFDEEEACAVFAP
Marasmius sp. AFHP31	PKSVITAMEIELLGFLDYDLRFDEEEACSMFAR
Mycena sanguinolenta	TLEDVNLMERQLIQLLDYDLRFDEAEACRYFAP
Guyanagaster necrorhizus MCA 3	QVKEVNLMEQQLLFLLDYDLRFDEEEACAAFGP
Marasmius tenuissimus	TVTEINLMEIQLLYLLDYDLRFSEEEACAIFAP
Marasmius tenuissimus	TLEDVNLMERQLIQLLDYDLRFDEAEACRYFAP
Amanita polypyramis BW_CC	TLEDVNLMERQLIQLLDYDLRFDEAEACRYFAP
Mycena indigotica	DVAEINLMEKQLLYLLDYDLRFSEDEICALFVP
Lentinula raphanica	QVGEVNLMEAQLLFLMDYDLRFNEEAACTTFAP
Infundibulicybe gibba	TIEEITLMETQLLGFLDFDLRFDEEEACRVFAP

Figure S2. Multiple sequence alignment of conserved amino acid regions outside the canonical cyclin_N domain in cyclin homologs identified by PSI-BLAST. Sequences were aligned using PSI-BLAST hits from *F. velutipes* cyclin genes and representative basidiomycete species.

	10 20 30
Mycena rebaudengoi	DVAEINLMEKQLLYLLDYDLRFDEAEACRLFAP
Mycena vulgaris	KVEEVNIMEKQLLFLLDYDLRFDEEEACAVFSP
Marasmius oreades	QVTEVNLMEQQLLYLLDYDLRFDEEEACTAFAP
Favolaschia claudopus	TLQNVNIMERQLLELLDYNLRFNEEEACRYFAP
Mycena capillaripes	QTHEVNLMEQQLLFLLDYDLRFDEEEACATFGP
Mycena citricolor	QVSEVNLMEQQLLFLLDYDLRFDEEEACAAFAP
Sphagnurus paluster	TLGEVNLMEQQLLALLDYDLRFDEEEACTTFAP
Mucidula mucida	DVAEINLMEKQLLFLLDYDLRFDEGETCTFFAP
Leucoagaricus sp. SymC.cos	KVDEINTMERELLRLLDFDMRFSEEEACLHFAP
Mycena pura	DVAEINLMEKQLLYLLGYDLRFDEMEAVKHFTP
Mycena floridula	PVKEVTLMEKQLLSLLDYDLRFDEEEACATFAP
Leucocoprinus birnbaumii	DLRQINVMEYQLLFILDYDLRFDELEACAYFAP
Plicaturopsis crispa FD-325 SS	DVAEINLMEKQLLYLLDYDLRFDEMEAIKHFSP
Favolaschia claudopus	DIAEINLMEKQLLFLLDYDLRFDELEACTLFAP
Hymenopellis radicata	QTHEVNLMEQQLLFLLDYDLRFDEEEACATFGP
Tricholomella constricta	KVAEINTMERELLRLLDFDMRFSEEEACLHFAP
Mycena chlorophos	DVAEINLMEKQLLFLLDYDLRFNEAETCRLFAP
Mycena chlorophos	QVSEVNLMETQLLFLLDYDLRFNEEAACTTFAP
Lentinula detonsa	QVSEVNLMETQLLFLLDYDLRFNEEAACTTFAP
Lentinula aff. detonsa	TIEEITLMETQLLGFLDYDLRFDEEEACRMFAP
Agaricus bisporus var. burnett	TIEEITLMETQLLGFLDYDLRFDEEEACRMFAP
Lentinula guzmanii	DVAEINLMEKQLLYLLDYDLRFDEEEAIKYFMP
Rhodocollybia butyracea	TIEEITLMETQLLGFLDYDLRFDEEEACRMFAP
Lentinula boryana	SLKEITLMETELLNFLDYDLRFDEEEACRTFAH
Phlebopus sp. FC_14	TIEEITLMETQLLGFLDYDLRFDEEEACRMFAP
Mucidula mucida	DLAEINLMEKQLLFLLDYDLRFDEREACKHFAP
Termitomyces sp. Mi166#008	KVDEINTMERELLRLLDFDMRFSEEEACLHFAP
Boletus coccyginus	DCPEINLMEKQLLYLLDYDLRFNEADACRLFAP
Lentinula raphanica	DLPEVNLMEKQLLFLLDYDLRFDEDEAVKHFAP
Agaricus bisporus var. burnett	TIEEITLMETQLLGFLDFDLRFDEEEACRVFAP
Gymnopilus junonius	DVAEINLMEKQLLYLLDYDLRFDEAEAIKYFMP
Lentinula raphanica	DVAEINLMEKQLLYLLDYELRFDEKEVCKHFAP
Lentinula raphanica	TIEEITLMETQLLGFLDFDLRFDEEEACRVFAP
Lentinula raphanica	TIEEITLMETQLLGFLDFDLRFDEEEACRVFAP
Tricholoma matsutake 945	TIEEITLMETQLLGFLDFDLRFDEEEACRVFAP
Amanita phalloides	NVSEINIMEGQLLYILDYDLRFTEAEACRAFSP
Suillus cothurnatus	DVAEINYMERQLLDLLDFDLRFNEAEACAIFAS
Amanita inopinata Kibby_2008	DLPEVNLMEEQLLYILGYDLRFDEHEACIHFAP
Leucogyrophana mollusca	DVAEINLMEKQLLYLLDYDLRFDEDEVCVLFDP
Flagelloscypha sp. PMI_526	DLAEINLMEKQLLFLLDYDLRFDELEACVHFSN
Agrocybe pediades	ELSEINLMEKQLLFLLDYNLRFTEQEALKSWAP
Termitomyces sp. Mn162	DVIEINLMEKQMLYLLDYDLRFDEAEVCKLFAP
Tricholoma furcatifolium	DCPEINLMEKQLLYLLDYDLRFNEADVCHLFAP
Lentinula aciculospora	DCPEINLMEKQLLYLLDYDLRFHEADACRLFAP
Pleurotus pulmonarius	TIEEITLMETQLLGFLDYDLRFDEEEACRVFAP

Figure S2. Continued.

	10 20 30
Boletus reticuloceps	QLDEINLMEKQLLFLLDYDLRFSEEDACTHFAP
Butyriboletus roseoflavus	DLAEVNLMEKQLLFLLDYDLRFDENEALKHFAP
Coniophora puteana RWD-64-598	DLAEVNLMEKQLLFLLDYDLRFDEDEAVTHFVP
Termitomyces sp. T159_Od127	DIAEINLMEKQLLFLLDYDLRFDEAEACTHFGP
Suillus cothurnatus	DAPEINLMEKQLLYLLDYDLRFNEADACRLFAP
Termitomyces sp. T32_za158	DLPEVNLMEEQLLYILGYDLRFDEHEACIHFAP
Hygrophoropsis aurantiaca	DAPEINLMEKQLLYLLDYDLRFNEADACRLFAP
Blastosporella zonata	DLAEINLMEKQLLFLLDYDLRFDELEACTHFQN
Suillus luteus UH-Slu-Lm8-n1	DGPEINLMEKQLLYLLDYDLRFNEADACRLFAS
Lentinula edodes	DLPEVNLMEEQLLYILGYDLRFDEHEACIHFAP
Pholiota molesta	TIEEITLMETQLLGFLDYDLRFDEEEACRMFAP
Flammula alnicola	DVPEINLMEKQLLYLLDYDLRFDEAEACKHFAP
Termitomyces sp. 'cryptogamus'	DVAEINLMEKQLLYLLDYDLRFDEAEACKHFAP
Suillus lakei	DCPEINLMEKQLLYLLDYDLRFNEADVCHLFAP
Suillus bovinus	DLPEVNLMEEQLLYILGYDLRFDEHEACIHFAP
Armillaria novae-zelandiae	DLPEVNLMEEQLLYILGYDLRFDEQEACIHFAP
Armillaria luteobubalina	KVTEINLMEIQLLYLLDYDLRFSEEEACTMFAP
Pleurotus djamor	KVTEINLMEIQLLYLLDYDLRFSEEEACAMFAP
Tephrocybe sp. NHM501043	EIPEINLMEKQLLFLLDYDLRFTEDEACAHFAP
Mycena rosella	DCPEINLMEKQLLYLLDYDLRFKEADACRLFAP
Suillus subluteus	QIKEVNLMEQQLLFLLDYDLRFDEEEACAAFAP
Gautieria morchelliformis	DLPEVNLMEEQLLYILGYDLRFDEHEACIHFAP
Suillus occidentalis	EMSEVNLMEKQLLSLLDYDLRFDEAEACRHFAP
Pholiota molesta	DLPEVNLMEEQLLYILGYDLRFDEHEACIHFAP
Scleroderma citrinum	DVPEINLMEKQLLYLLDYDLRFDEAEACKHFAP
Scleroderma yunnanense	DIAEVNLMEKQLLFLLDYDLRFNEEEACEHFAP
Pleurotus djamor	DIAEVNLMEKQLLFLLDYDLRFNEEEACEHFAP
Lentinula aff. lateritia	EIPEINLMEKQLLFLLDYDLRFTEDEACAHFAP
Lentinula lateritia	TIEEITLMETQLLSFLDYDLRFDEEEACRMFAP
Lentinula edodes	TIEEITLMETQLLGFLDYDLRFDEEEACRMFAP
Lentinula novae-zelandiae	TIEEITLMETQLLGFLDYDLRFDEEEACHMFAP
Boletus edulis	TIEEITLMETQLLGFLDYDLRFDEEEACRMFAP
Chiua virens	DLAEVNLMEKQLLFLLDYDLRFDENEALKHFAP
Suillus clintonianus	DLSEVNLMEKQLLFLLDYDLRFAEDEALKHFAP
Suillus discolor	DLPEVNLMEEQLLYILGYDLRFDEHEACIHFAP
Suillus brevipes Sb2	DLPEVNLMEEQLLYILGYDLRFDEQEVCIHFAP
Panaeolus cyanescens	DLPEVNLMEEQLLYILGYDLRFDEHEACIHFAP
Suillus subalutaceus	DVAEINLMEKQLLYLLDYDLRFDEEQACTHFAP
Amanita rubescens	DLPEVNLMEEQLLYILGYDLRFDEHEACIHFAP
Arthromyces matolae	SVDEINVMEKQLLSLLNYDLRFDEAEVCAIFAP
Suillus hirtellus	DNPEINLMEKQLLYLLDYDLRFSEADACRLFAP
Mycena crocata	DLPEVNLMEEQLLYILGYDLRFDEQEVCIHFAP
Gymnopilus dilepis	QVNEVNLMEQQLLFLLDYDLRFDEEEACAAFAP
Somion occarium	DVAEINLMEKQLLYLLDYELRFNEEEICNHFAP
Suillus paluster	DPAEINLMEKQLLFLLDYDLRFNEQEAIAHFSP

Figure S2. Continued.

	10 20 30
Psilocybe cyanescens	DLPEVNLMEEQLLYILGYDLRFDEREACTHFAV
Suillus tomentosus	DVAEINLMEKQLLYLLDYELRFDEEEVCKLFAP
Suillus plorans	DLPEVNLMEEQLLYILGYDLRFDEQEVCIHFAP
Agrocybe pediades	DLPEVNLMEEQLLYILGYDLRFDEQEVCIHFAP
Suillus variegatus	DVIEINLMEKQLLYLLDYDLRFDEAEVCKLFAP
Suillus placidus	DLPEVNLMEEQLLYILGYDLRFDEQEVCIHFAP
Lanmaoa asiatica	DLPEVNLMEEQLLYILGYDLRFDEHEACIHFAP
Suillus fuscotomentosus	DLAEVNLMEKQLLFLLDYDLRFDEDEAVKHFVP
Mycena filopes	DLPEVNLMEEQLLYILGYDLRFDEQEVCIHFAP
Hohenbuehelia grisea	KTSEVNLMEQQLLFLLDYDLRFDEEEACTAFAP
Schizophyllum amplum	DLSEINLMEKQLLFLLDYDLRFSEEEACGHFAP
Volvariella volvacea WC 439	SCPEINLMEQQLLFLLDYDLRFDEKTTLDVWAP
Abortiporus biennis	DVAEVNLMEKQLLYLLDYDLRFNEKEACTHFAP
Abortiporus biennis	DCAEINLMEKQLLFLLDYDLRFDEKEAIAHFST
Armillaria mellea	DCAEINLMEKQLLFLLDYDLRFDEKEAIAHFST
Paxillus ammoniavirescens	NVTEINLMEIQLLYLLDYDLRFSEEEACALFAP
Mycena leptocephala	DLAEVNLMEKQLLFLLDYDLRFDEQEAIKHFAP
Suillus weaverae	QVTEVNLMEQQLLFLLDYDLRFDEEEACAAFAP
Cyclocybe aegerita	DLPEVNLMEEQLLYILGYDLRFDEHEACIHFAP
Cristinia sonorae	DVAEINLMEKQLLYLLDYDLRFNEEEVCKRFAP
Mycena sanguinolenta	DPAEINLMEKQLLFLLDYDLRFDENEALLHFAP
Paxillus rubicundulus Ve08.2h1	QVKEVNLMEQQLLFLLDYDLRFDEEEACVAFGP
Pleurotus pulmonarius	DLAEVNLMEKQLLFLLDYDLRFDEQEAIKHFAP
Pleurotus pulmonarius	QLDEINLMEKQLLFLLDYDLRFSEEDACTHFAP
Marasmius crinis-equi	QLDEINLMEKQLLFLLDYDLRFSEEDACTHFAP
Sparassis crispa	SLEDVNLMERQLIQLLDYNLRFDEAEACRYFAP
Rhizopogon vesiculosus	DLKEINLMEMQMLYLLDYDLRFSEQEALAQFSA
Pisolithus microcarpus	DLPEVNLMEEQLLYILGYDLRFDEREACIHFAP
Pleurotus eryngii	DLAEVNLMEKQLLSLLEYDLGFDEEEACKFFAP
Amanita thiersii Skay4041	QLDEINLMEKQLLFLLDYDLRFSEEDACTHFAP
Grifola frondosa	DVAEINLMEKQLLYLLDYDLRFDEAEACVQFSP
Pleurotus ostreatus PC15	DLAEINLMEKQLLFLLDYDLRFEEQEALVHFAP
Dentipellis fragilis	QLDEINLMEKQLLFLLDYDLRFSEEDACTHFAP
Peniophora sp. CBMAI 1063	DISEINLMEKQFLFLLDYDLRFDEAEACNFFAP
Rhodonia placenta	DINEINLMEKQLLFLLDYELRFDENEALAAFEP
Lyophyllum atratum	DLAEINLMEKQLLFLLDYDLRFDELEAILHFAP
Phlebia brevispora	DVAEINLMETQLLYLLNYDLRFHEAETCRLFSP
Suillus spraguei	DPAEINLMEKQLLFLLDYDLRFDEAEAIKHFTP
Schizophyllum commune	DLPEVNLMEEQLLYILGYDLRFDEHEACIHFAP
Cytidiella melzeri	STPEINLMEQQLLYLLDYDLRFDETTALNVWTP
Mycena alexandri	EPSEINLMEKQLLFLLDYDLRFEEAEAISHFAP
Athelia psychrophila	KTSEVNLMERQLLFLLDYDLRFDEEEACAAFAP
Schizophyllum commune	EIAEINLMERQLLYFLDFDLRFDEKEACAHFAP
Schizophyllum commune Tattone	STPEINLMEQQLLYLLDYDLRFDETTALNVWTP
Obba rivulosa	STPEINLMEQQLLYLLDYDLRFDETTALNVWTP

Figure S2. Continued.

	10 20 30
Schizophyllum commune Loenen D	DLAEINLMEKQLLFLLDYDLRFDEQEALIYFAP
Pisolithus albus	STPEINLMEQQLLYLLDYDLRFDETAALNVWTP
Hermanssonia centrifuga	DLAEVNLMEKQLLSLLDYDLGFDEEEACKFFAP
Mycena amicta	EPAEINLMEKQLLFLLDYDLRFDESEAMAHFAP
Leucocoprinus leucothites	QVSEVNLMESQLLFLLDYDLRFDEEAACASFAP
Lentinula edodes	DVAEINLMEKQLLYLLDYDLRFDEMEAVKHFTP
Dentipellis sp. KUC8613	TIEEITLMETQLLGFLDYDLRFDEEEACHMFAP
Schizophyllum commune	DISEINLMEKQFLFLLDYDLRFDEAEACNFFAP
Hydnomerulius pinastri MD-312	STPEINLMEQQLLYLLDYDLRFDETTALNVWTP
Schizophyllum commune	DIAEINLMEKQLLFLLDYDLRFDEQEASSHFAP
Asterophora parasitica	STPEINLMEQQLLYLLDYDLRFDETTALNVWTP
Laccaria amethystina LaAM-08-1	DVAEINLMEKQLLFLLDYDLRFNEADTCRLFAP
Schizophyllum fasciatum	DVNEINLMEKQLLYLLDYDLRFFEPEVCALFAP
Schizophyllum commune	STPEINLMEQQLLYLLDYDLRFDEATALHIWTP
Amylocystis lapponica	STPEINLMEQQLLYLLDYDLRFDETTALNVWTP
Roridomyces roridus	DLAEINLMEKQLLFLLDYDLRFSEQEALASFAP
Schizophyllum commune	QVHEVNLMEQQLLFLLDYDLRFQEEEACSAFAP
Serpula lacrymans var. lacryma	STPEINLMEQQLLYLLDYDLRFDETTALNVWTP
Phanerochaete sordida	DLAEINLMEKQLLFLLDYDLRFDEAEACIHLAS
Moniliophthora perniciosa FA55	GTDEINLMEKQLLFLLDYDLRFDEAEAIQHFAP
Athelia sp. TMB	PVSEINLMERQLLTLLDYDLRFDEEEACRYFAP
Panaeolus papilionaceus	EIAEINLMERQLLFFLDYDLRFDEEEACTFFAP
Schizophyllum commune H4-8	DVAEINLMEKQLLYLLDYDLRFDEEQACTHFAP
Artomyces pyxidatus	STPEINLMEQQLLYLLDYDLRFDETTALNVWTP
Laccaria bicolor S238N-H82	DIAEINLMERQLLFLLDYDLRFDETEALAHFAP
Lentinus tigrinus ALCF2SS1-7	DVNEVNLMEKQLLYLLDYDLRFFEPEVCALFAP
Ramaria rubella	ELAEVNLMEKQLLFLLNYDLRFDEDEALLHFAP
Phlegmacium glaucopus	EMTEINLMEKQLLYLLDYDLRFDEAEACRHFAP
Pleurotus cornucopiae	DVAEINLMEKQLLYLLDYDLRFDELEACTAFAP
Taiwanofungus camphoratus	QLDEINLMEKQLLFLLDYDLRFSEEDACAHFAP
Melanogaster broomeanus	DLAEINLMEKQLLYLLDYDLRFDEQAAVNHFSV
Suillus subaureus	DLAEVNLMEMQLLFILGYELRFDEQEAIIHFAP
Trametes versicolor	DLPEVNLMEEQLLYILGYDLRFDEHEACIHFAP
Mycena albidolilacea	ELAEVNLMEKQLLYLLNYDLRFDEEEALRHFAP
Mycena olivaceomarginata	QVTEVNLMEQQLLFLLDYDLRFDEEEACAAFGP
Lentinus brumalis	QVTEVNLMEQQLLFLLDYDLRFDEEEACAAFGP
Lentinula edodes	ELAEVNLMEKQLLFLLNYDLRFNEEEALLHFAP
Collybiopsis luxurians FD-317	TIEEITLMETQLLGFLDYDLRFDEEEACRMFAP
Pilatotrama ljubarskyi	TIEEITLMETQLLGFLDYDLRFDEEEACRMFAP
Suillus americanus	ELAEVNLMEKQLLYLLNYDLRFDEEEALLHFAP
Lactarius quietus	DLPEVNLMEEQLLYILGYDLRFDEHEACIHFAP
Physisporinus lineatus	HLHEINLMERELLAMLDYDLRFDEAEACRLFEP
Suillus decipiens	DPVEINLMEKQLLFLLDFDLRFEEKDAIAHFAP
Lactarius indigo	DLPEVNLMEEQLLYILGYDLRFDEHEACIHFAP
Mycena venus	HLHEINLMERELLAMLDYDLRFDEAEACRLFEP

Figure S2. Continued.

	10 20 30
Hericium alpestre	QVTEVNLMEQQLLFLLDYDLRFDEEEACAAFGP
Pleurotus ostreatus	DISEINLMEKQLLFLLDYDLRFDEHDACTHFAP
Taiwanofungus camphoratus	QLDEINLMEKQLLFLLDYDLRFSEEDACTHFAP
Pisolithus microcarpus 441	DLAEINLMEKQLLYLLDYDLRFDEQAAVNHFSV
Thelephora terrestris	DLAEVNLMEKQLLSLLEYDLGFDEEEACKFFAP
Pisolithus marmoratus	GVFEITRTEVELLAILDYDLRFDEAETRYHFAP
Phanerochaete carnosa HHB-1011	DLAEVNLMEKQLLSLLDYDLDFDEEEACKHFAP
Phellinidium pouzarii	DPAEINLMEKQLLFLLDYDLRFDETEATNHFAP
Dendrothele bispora CBS 962.96	ENAEINLMESQLIFLLDFDLRFSEEQAIEHWAP
Gyrodon lividus	PLKEINLMESQLLSMLDFDLRFDEEEACRVFAP
Imleria badia	DLAEVNLMEKQLLFLLDYDLRFDEQEAIKHFAP
Lactarius deliciosus	DLAEVNLMEKQLLFLLDYDLRFDEDEAVKHFAP
Epithele typhae	HLHEINLMERELLAMLDYDLRFDEAEACRLFEP
Rhizopogon vinicolor AM-OR11-0	ELAEVNLMEKQLLFLLNYDLRFDEDEALEFFAP
Agaricus bisporus var. bisporu	DLSEVNLMEEQLLYILGYDLRFDEREACAHFAP
Pisolithus thermaeus	DVAEINLMEKQLLYLLDYDLRFDEEEAIKYFMP
Irpex lacteus	DLAEVNLMEKQLLSLLDYDLDFDEEEACKFFAP
Irpex rosettiformis	DPSEINLMEKQLLFLLDYDLRFDEAEAISHFAP
Agrocybe chaxingu	DPSEINLMEKQLLFLLDYDLRFDEAEAIVHFAP
Pisolithus croceorrhizus	DVAEINLMEKQLLYLLDYDLRFNEEEVCKLFVP
Russula earlei	DLAEVNLMEKQLLSLLDYDLDFDEEEACKFFAP
Pisolithus tinctorius	DLAEINLMEKQLLFLLDYDLRFDELEACRLFEP
Trametopsis cervina	DLAEVNLMEKQLLFLLDYDLDFDEEEACKHFAP
Laetiporus sulphureus 93-53	EPSEINLMEKQLLFLLDYDLRFDEAEAIRHFAP
Astraeus odoratus	DLAEINLMEKQLLFILDYDLRFDEPEAILHFAP
Pterulicium gracile	DLAEVNLMEKQLLFLLDYDLGFDEEEACRYFAP

Figure S2. Continued.
