# Supplementary material for: High Carbon Dioxide Concentration Inhibits Pileus Growth of Flammulina velutipes by Downregulating Cyclin Gene Expression
Source: J Fungi (Basel). 2025 Jul 24;11(8):551. doi: 10.3390/jof11080551 (PMC12387923; doi:10.3390/jof11080551)
Supplement: Supplementary file 1 [file jof-11-00551-s001.zip › Figure S3.pdf]

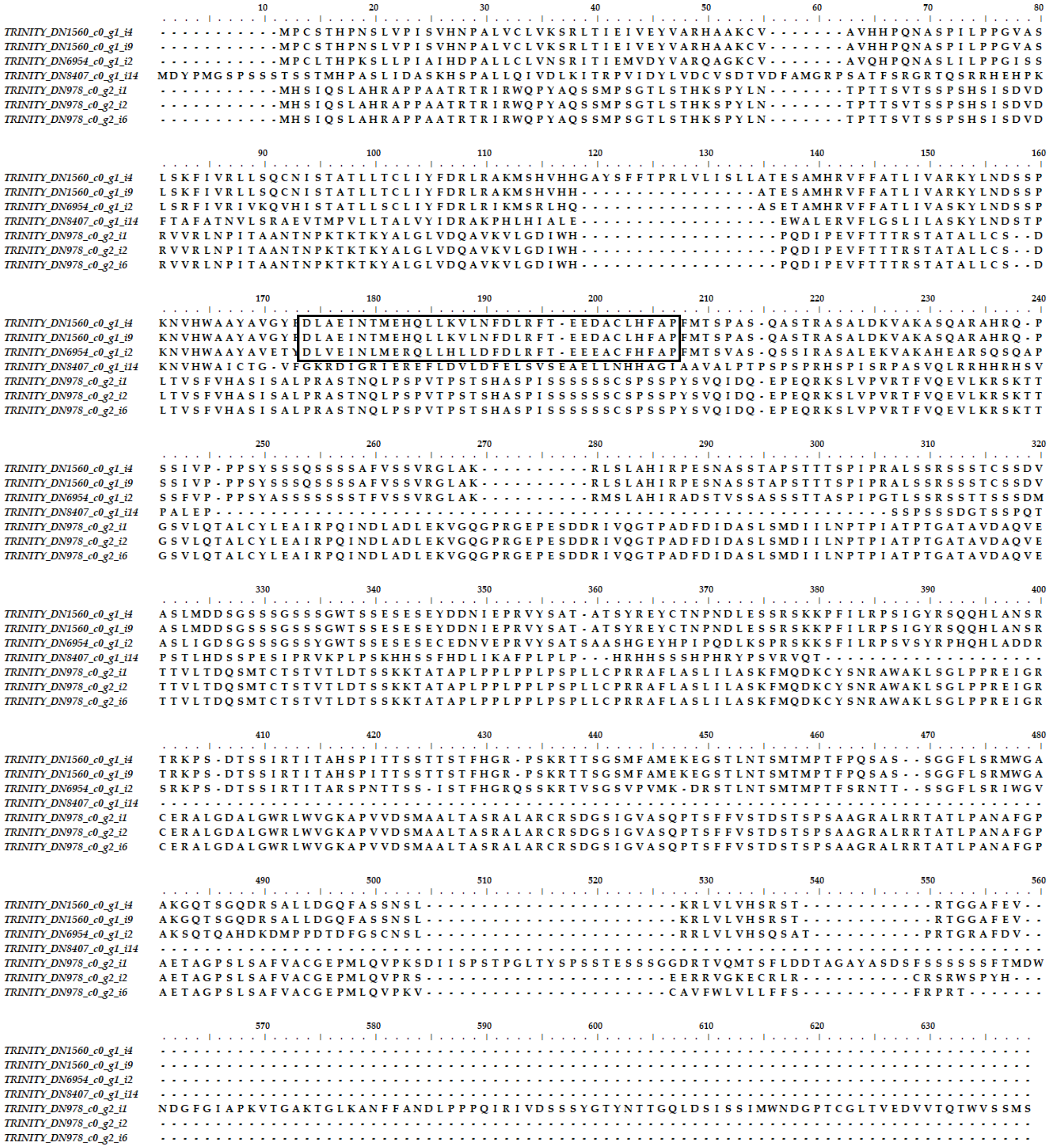

Figure S3. Comparative sequence analysis of PHO80-like cyclin genes within the *F. velutipes* genome. Boxes indicate the specific conserved sequence regions that are commonly shared with Basidiomycota homologs.
