# Supplementary material for: High Carbon Dioxide Concentration Inhibits Pileus Growth of Flammulina velutipes by Downregulating Cyclin Gene Expression
Source: J Fungi (Basel). 2025 Jul 24;11(8):551. doi: 10.3390/jof11080551 (PMC12387923; doi:10.3390/jof11080551)
Supplement: Supplementary file 1 [file jof-11-00551-s001.zip › Figure S4.pdf]

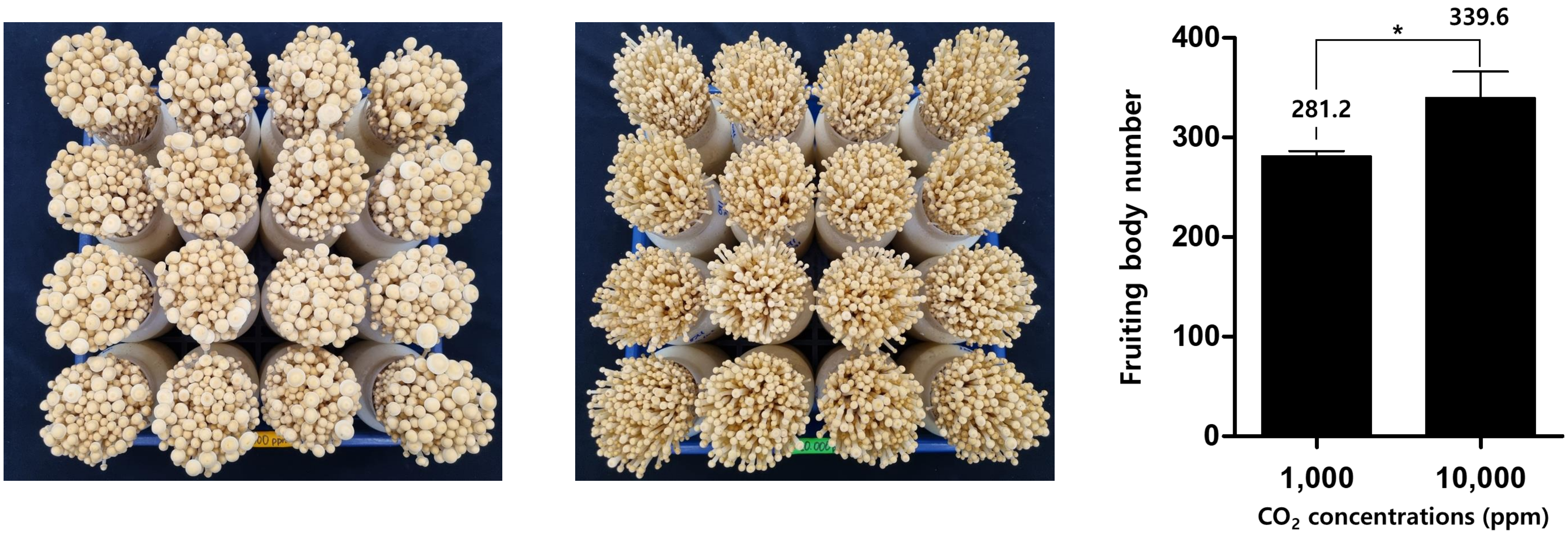

(a) (b)

Figure S4. Comparison of fruiting body numbers in *F. velutipes* under 1,000 ppm and 10,000 ppm CO_2. Representative images of culture bottles grown under 1,000 ppm CO_2 (left) and 10,000 ppm CO_2 (right) (a). Average number of fruiting bodies per bottle under each condition (b). Asterisks indicate statistically significant differences between treatments ($P < 0.05$; Student's *t*-test).
